# Islet Amyloid Polypeptide: A Partner in Crime With Aβ in the Pathology of Alzheimer's Disease

**DOI:** 10.3389/fnmol.2020.00035

**Published:** 2020-03-20

**Authors:** Ana F. Raimundo, Sofia Ferreira, Ivo C. Martins, Regina Menezes

**Affiliations:** ^1^iBET - Instituto de Biologia Experimental e Tecnológica, Oeiras, Portugal; ^2^CEDOC - Chronic Diseases Research Center, Faculdade de Ciências Médicas, Universidade Nova de Lisboa, Lisbon, Portugal; ^3^ITQB-NOVA, Instituto de Tecnologia Química e Biológica António Xavier, Universidade Nova de Lisboa, Oeiras, Portugal; ^4^Instituto de Medicina Molecular, Faculdade de Medicina, Universidade de Lisboa, Lisbon, Portugal

**Keywords:** Aß-42, Alzheimer's disease, amylin, diabetes, IAPP, protein aggregation

## Abstract

Diabetes affects hundreds of millions of patients worldwide. Despite the advances in understanding the disease and therapeutic options, it remains a leading cause of death and of comorbidities globally. Islet amyloid polypeptide (IAPP), or amylin, is a hormone produced by pancreatic β-cells. It contributes to the maintenance of glucose physiological levels namely by inhibiting insulin and glucagon secretion as well as controlling adiposity and satiation. IAPP is a highly amyloidogenic polypeptide forming intracellular aggregates and amyloid structures that are associated with β-cell death. Data also suggest the relevance of unprocessed IAPP forms as seeding for amyloid buildup. Besides the known consequences of hyperamylinemia in the pancreas, evidence has also pointed out that IAPP has a pathological role in cognitive function. More specifically, IAPP was shown to impair the blood–brain barrier; it was also seen to interact and co-deposit with amyloid beta peptide (Aß), and possibly with Tau, within the brain of Alzheimer's disease (AD) patients, thereby contributing to diabetes-associated dementia. In fact, it has been suggested that AD results from a metabolic dysfunction in the brain, leading to its proposed designation as type 3 diabetes. Here, we have first provided a brief perspective on the IAPP amyloidogenic process and its role in diabetes and AD. We have then discussed the potential interventions for modulating IAPP proteotoxicity that can be explored for therapeutics. Finally, we have proposed the concept of a “diabetes brain phenotype” hypothesis in AD, which may help design future IAPP-centered drug developmentstrategies against AD.

## Introduction

Amyloidogenesis is a process by which peptides spontaneously self-assemble into higher order structures, namely oligomers, protofibrils, and mature amyloid fibrils (Martins et al., 2008; Maurer-Stroh et al., 2010; Hauser et al., 2014). These mature amyloid fibrils are highly ordered structures with fibrillar aggregates derived from different amyloidogenic amino acid sequences that share common features (Maurer-Stroh et al., 2010). The current consensus is that the amyloid fibrils are not the main cause of toxicity (Martins et al., 2008; Kuperstein et al., 2010; Hauser et al., 2014). This seems to be mostly down to precursor oligomers and protofibrils, which are associated with a number of the so-called amyloid diseases, including type 2 diabetes mellitus (T2DM), Alzheimer's disease (AD), Parkinson's disease, and cataracts (Hauser et al., 2014; Cremades and Dobson, 2018).

T2DM, the most prevalent type of diabetes, is an islet amyloid polypeptide (IAPP)-associated pathology (Cukierman et al., 2005; Westermark et al., 2011; Yang and Song, 2013). Dementia also represents a major public concern, affecting 50 million people worldwide. AD, the most common form of dementia in North America (Alzheimer's Association, 2016; Bondi et al., 2017; Lane et al., 2018), is associated with amyloid beta peptide 42 (Aß-42) (Martins et al., 2008; Kuperstein et al., 2010). The amyloid hypothesis on AD pathology is, however, called into question by the undeniable role of Tau aggregation and other important players, as has been reviewed (Makin, 2018).

There is much evidence to support the close association between T2DM and AD. IAPP (also known as amylin) and Aß-42 were proven to co-deposit, contributing to AD onset and progression (Jackson et al., 2013; Wijesekara et al., 2017). In addition, it the molecular interaction between Tau and IAPP was recently proved (Arya et al., 2019). At last, AD is associated with insulin resistance and an imbalance of glucose levels in the brain (Cukierman et al., 2005; Yang and Song, 2013), earning the designation of type 3 diabetes (T3DM) (de la Monte, 2014; Kandimalla et al., 2017; Leszek et al., 2017). Given these links, we have reviewed the mechanisms of IAPP dysfunction in diabetes and dementia, particularly in AD, thus adding to the recent view of multi-factorial contributions to both diseases. Furthermore, we have also discussed the potential interventions for modulating IAPP proteotoxicity that can be explored for therapeutics, encouraging new venues for treatment.

## IAPP and Diabetes

Diabetes mellitus (DM) is one of the major causes of premature illness and mortality worldwide (Federation, 2009). High blood glucose levels and glucose intolerance, as a consequence of a defective insulin production/secretion by pancreatic β cells (β-cells) or insulin sensitivity (Stumvoll et al., 2005; Tan et al., 2019), are the typical clinical features of the disease. In T2DM, impairment and loss of β-cell mass has been associated with diverse pathological phenomena, including glucolipotoxicity, islet cholesterol accumulation, and islet inflammation (Poitout and Robertson, 2002; Ishikawa et al., 2008; Brunham et al., 2010; Donath and Shoelson, 2011). Equally important are the current views that regard IAPP dyshomeostasis, intracellular accumulation of IAPP oligomers, and IAPP amyloid deposition in the islets of Langerhans as detrimental events in β-cell dysfunction and disease (Kanatsuka et al., 2018).

**Figure 1:**
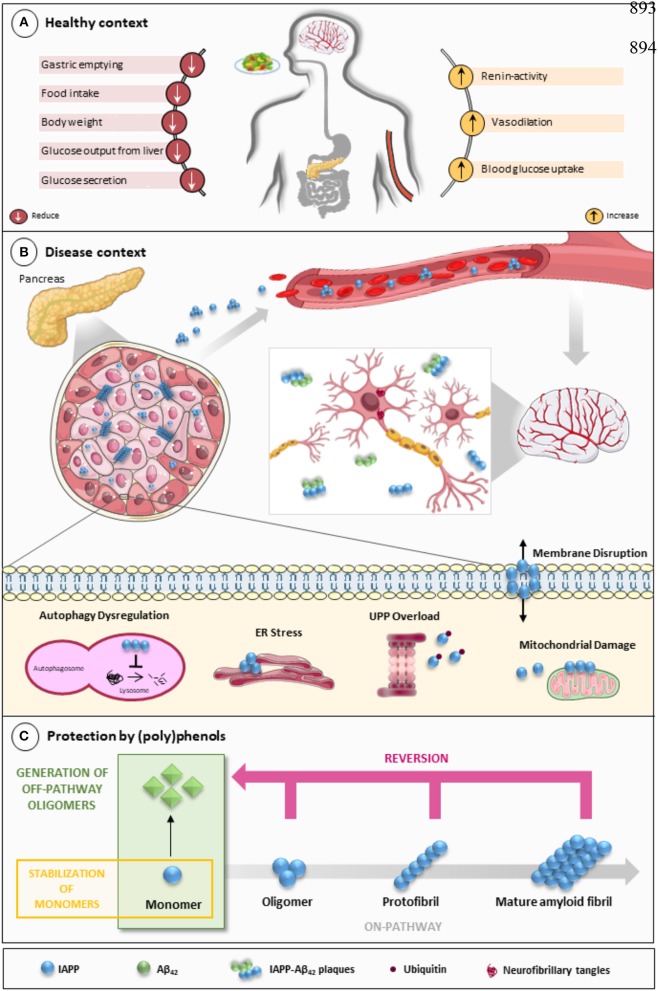
IAPP on physiological and pathological contexts and (poly)phenols-mediated protection. **(A)** In healthy conditions, IAPP is co-secreted with insulin to regulate glucose metabolism and homeostasis in a post-meal condition. Several functions are attributed to IAPP: slowing down gastric emptying, thereby reducing food intake and body weight; reducing glucose output from liver and glucagon secretion; and stimulating the renin-angiotensin system, vasodilation, and blood glucose uptake. **(B)** In disease conditions, IAPP pathological species deposit in the pancreas and in brain microvasculature where they induce the injury of small vessels and reach the brain parenchyma. In the brain environment, IAPP forms heterogeneous deposits with Aβ molecules increasing neurotoxicity. Proteostasis imbalance caused by Aβ/IAPP and tau may promote a set of molecular changes that culminate in glucose homeostasis dysregulation, cell death, and neurodegeneration. The molecular pathways of β-cell dysfunction are depicted: autophagy dysregulation; ER stress; UPP overload; membrane instability; and mitochondrial damage. **(C)** Protection mediated by (poly)phenols is associated with the stabilization of IAPP monomers, the remodeling of amyloids, protofibrils, and toxic oligomers to non-fibrillogenic “off-pathway” oligomers and monomers. Aβ, Amyloid beta; ER, Endoplasmic Reticulum; IAPP, Islet Amyloid Polypeptide; Ub, Ubiquitin; UPP, Ubiquitin Proteasome Pathway.

IAPP is a 37-amino acid neuroendocrine hormone that plays an important role in regulating metabolism and glucose homeostasis (Figure 1A). In circulation, IAPP and insulin act as synergistic partners: they stimulate the uptake of blood glucose into muscle and fat tissues and inhibit the endogenous glucose output from the liver, thus stabilizing the blood sugar levels in post-meal conditions (Zhang et al., 2016). Physiologically, IAPP also reduces the secretion of nutrient-stimulated glucagon, regulates gastric emptying and satiation (Lutz, 2010; Akter et al., 2016), and regulates blood pressure while having an effect on the renin-angiotensin system (Wookey et al., 1998).

IAPP and insulin are co-secreted and processed by proprotein convertase (PC) 1/3, PC 2, and carboxypeptidase E (Yonemoto et al., 2008). During its biogenesis, IAPP is synthesized as an 89-residue preprohormone (Sanke et al., 1988). Its signal peptide is cleaved throughout the transport into the endoplasmic reticulum (ER) to form proIAPP (Akter et al., 2016), which is then processed in the late Golgi complex. To yield the mature active form of the hormone, IAPP suffers amidation of the C-terminal end, and a disulphide bond is formed between cysteines at positions two and seven (Westermark et al., 2011; Akter et al., 2016; Bower and Hay, 2016). Once produced, mature IAPP is co-packaged with insulin in secretory granules of β-cells to then be co-released in response to glucose (Kahn et al., 1993; Gedulin et al., 1997; Zhang et al., 2016). In a pre-diabetes/diabetes phenotypes, the increased production of insulin is accompanied by augmented IAPP levels (Kahn et al., 1991; Mulder et al., 1996). The overload and impairment of β-cell processing machinery leads to the accumulation of unprocessed IAPP forms (Westermark et al., 2000; Paulsson et al., 2006). These events, together with the overwhelming of the ER, generate a feed-forward cycle that promotes IAPP oligomerization, fibril formation, and β-cell injury. Elevated proIAPP levels and amyloid deposition in β-cells lacking PC1/3 and PC2 (Marzban et al., 2006), as well as the presence of proIAPP in intracellular fibrils (Paulsson et al., 2006), corroborate this idea. Despite this, the role of unprocessed IAPP forms in the disease is not fully understood.

Under pathological conditions, increased IAPP expression and the generation of aberrant IAPP intermediates favor misfolding, which leads to the formation of toxic aggregates through a seeding-nucleation model, similar to prion replication (Mukherjee et al., 2017). As misfolded molecules accumulate, they build up into intracellular oligomers and larger amyloid fibrils, which deposit in surrounding tissues, thus disrupting the normal islet architecture and functioning (Zhang et al., 2016). Deposits of aggregated IAPP are present in the pancreas of about 90% of T2DM patients, thus representing a histopathological hallmark of the disease (Westermark and Grimelius, 1973; Mukherjee et al., 2017). Corroborating the toxicity of these aggregates in diabetes, the IAPP allele S20G, which raises IAPP aggregation propensity (Sakagashira et al., 2000), has been associated with premature onset diabetes and has accelerated the decline of endogenous insulin secretion when compared to non-S20G T2DM individuals (Morita et al., 2011). Moreover, a transgenic mice model expressing human IAPP (hIAPP) spontaneously developed amyloidosis, showing impaired insulin production, β-cell loss, and fasting hyperglycemia (Janson et al., 1996).

Although the link between IAPP aggregation and β-cell loss seems to be convincing, there are some questions that remain poorly understood, including (a) the initiation site and triggers of amyloid formation, (b) the mechanisms of IAPP-mediated toxicity in β-cell death, and (c) the nature of toxic IAPP species (Kanatsuka et al., 2018). Initially, mature amyloid fibrils were presumed to be the pathological structures (Lorenzo and Yankner, 1996), however, the current consensus is that toxicity is mostly associated with soluble oligomers and protofibrils, which may act as the trigger agents for β-cell depletion and diabetes onset (Haataja et al., 2008; Zhao et al., 2009; Zhang et al., 2016).

Oligomeric IAPP species form ion-leaking pores in the cell membranes (Gurlo et al., 2010; Li et al., 2016b), leading to enhanced membrane fluidity, calcium dysregulation, and decreased cell viability (Huang et al., 2010). IAPP oligomers have also been found within disturbed mitochondrial membranes in transgenic hIAPP mice and T2DM patients (Gurlo et al., 2010). Unstable mitochondrial membrane potential induced by toxic oligomers is thought to be involved in the overproduction of reactive oxygen species (ROS), which are currently considered to be potential initiators of IAPP toxicity (Konarkowska et al., 2005). ER stress and impairment of proteasome function have also been associated with hIAPP-induced toxicity (Casas et al., 2007; Gurlo et al., 2010), however, in studies with cultured islets producing IAPP at more physiological levels, ER stress was not detected (Hull et al., 2009).

In heterozygous *hIAPP*+ mice with β cell–specific *Atg7* deficiency (*hIAPP*^+^*Atg7*^Δ*βcell*^ mice), the accumulation of toxic oligomers, the loss of β-cells, and diabetes development is linked to autophagy disruption, and this is suggestive of a role for autophagy in IAPP toxicity (Kim et al., 2014). Moreover, inhibition of lysosomal degradation in HIP (hIAPP transgenic) rats increases hIAPP-mediated toxicity, whereas autophagy stimulation protects β-cells against hIAPP-induced apoptosis (Rivera et al., 2011). Chronic inflammation is also observed in local and systemic amyloidosis due to the activation of the NLRP3 inflammasome by hIAPP aggregates (Masters et al., 2010). A general view of IAPP pathological mechanisms is given in Figure 1B.

## IAPP Pathology in the Brain

AD was considered for a long period to be caused by Aβ amyloidogenesis and/or Tau aggregation (Makin, 2018). Indeed, the presence of extracellular Aβ-42 amyloid plaques and intracellular aggregates of hyperphosphorylated Tau are the classical diagnostic markers of the disease (Glenner et al., 1984; Gotz, 2001; Gong et al., 2003). Aβ exists mainly in two forms, Aβ-40 and Aβ-42, composed of 40 and 42 amino acids, respectively, and the increase of the Aβ-42/Aβ-40 ratio is strongly correlated with AD severity (Kuperstein et al., 2010). Given the importance of these players in disease pathophysiology, AD research has been so focused on them that other possible agents have been somewhat overlooked.

More recently, IAPP has emerged as a novel player in AD pathology (de la Monte and Wands, 2008; Wijesekara et al., 2017; Norwitz et al., 2019; Qiu et al., 2019). Notwithstanding, the mechanisms by which IAPP contributes to AD pathology are still unclear and deserve further enquiry. It is known that IAPP and Aβ interact with each other and that IAPP promotes Aβ aggregation in a seeding-like manner, leading to the formation of cross-seeded oligomers (Andreetto et al., 2010; Rezaei-Ghaleh et al., 2011; Yan et al., 2014; Hu et al., 2015; Bakou et al., 2017; Moreno-Gonzalez et al., 2017; Ge et al., 2018; Armiento et al., 2019). Interestingly, an aggregation blocker mimicking IAPP has been proven to work against Aβ (Yan et al., 2007).

Hyperamylinemia has been pointed out as a possible trigger for IAPP misfolding and aggregation, which may cause damage in the brain (Jackson et al., 2013) and other organs by various mechanisms that include the toxic gain-of-function of IAPP aggregates and the loss of IAPP physiological functions (Westermark et al., 2011; Despa et al., 2012, 2014). In addition, IAPP dyshomeostais may affect other organs, particularly the brain, in Aβ-42-dependent and -independent manners. This is illustrated by studies showing that IAPP deposition impairs brain function regardless of Aβ-42 pathology (Srodulski et al., 2014) and that the brain of AD patients can also have IAPP deposits, alone or in the presence of Aβ-42 (Fawver et al., 2014), even if clinical signs of diabetes are absent (Jackson et al., 2013; Oskarsson et al., 2015). A remarkable aspect is the fact that the IAPP analog pramlintide is able to have a neuroprotective effect, both in AD pathogenesis as well as on cognition in general (Adler et al., 2014). This is in line with observations that the key regions involved in Aβ-42-IAPP interaction—the interface amino acid residues—are at the same time high-affinity binding sites in both the cross- and self-aggregation of these molecules (Andreetto et al., 2010). Pramlintide possibly modulates these interactions by preventing them or promoting the formation of biologically inactive fibrils. However, the *in silico* cross seeding of Aβ-42 and IAPP fibril-like oligomers still needs to be complemented with further experimental evidence to support this hypothesis (Berhanu et al., 2013). In addition to Aβ-42, it was also reported that the major component of cerebrovascular plaques in the AD brain, the Aβ-40, can cross-seed IAPP fibrillization, suggesting that these two peptides might populate states that cross-interact (O'Nuallain et al., 2004). Other mechanisms by which IAPP dyshomeostasis exacerbates Aβ-42 toxicity in the brain may include ROS generation (Jhamandas and MacTavish, 2004; Lim et al., 2010) and the breakdown of insulin degrading enzyme activity, which is responsible for insulin, IAPP, and Aβ degradation (Kurochkin and Goto, 1994; McDermott and Gibson, 1997).

As IAPP produced in the pancreas was shown to cross the blood–brain barrier (Banks et al., 1995; Banks and Kastin, 1998) and to act on brain receptors, another important aspect of IAPP pathophysiology in the brain is its role in neuronal network function. Therefore, the effects of IAPP on neuronal and glial cells have been investigated (Chaitanya et al., 2011; Xi et al., 2019). As the primary site of IAPP action, the *area postrema* (AP) is the brain structure best characterized in terms of IAPP effects. While IAPP was shown to promote the formation of AP neuronal projections in neonatal rodents, in adult Wistar rats, IAPP injections were reported (1) to affect genes controlling neurogenesis, particularly *NeuroD1*, (2) to increase the number of newly proliferated AP*-*cells, and (3) to promote differentiation of these cells into neurons (Liberini et al., 2016). A study to investigate the mechanism by which IAPP modulates neuronal excitability in AP neurons in rat brainstem slices revealed that IAPP induced changes in excitatory responses of neurons not displaying the hyperpolarization-activated cation current. Furthermore, this study revealed that IAPP receptors were mainly located on presynaptic glutamatergic terminals connecting these neurons and that IAPP can increase glutamate release enough to cause cell firing (Fukuda et al., 2013). Likewise, hIAPP was shown to cause a dose-dependent membrane depolarization and an increase in firing frequency in neurons of the diagonal band of Broca, a cholinergic basal forebrain nucleus, in rats (Li and and Li, 2012). Hence, IAPP dysregulation may have important implications in neuronal function. IAPP receptors were also proven to be mediators of the deleterious actions of Aβ-42 in human neurons (Jhamandas et al., 2011). In this sense, amylin receptors are seen as potential targets for AD therapies (Fu et al., 2017).

AD is also considered a metabolic disease to a large extent. It is clear that the brain loses its capacity to deal with glucose and to respond to insulin and insulin-like growth factor (IGF) (Rivera et al., 2005; Liu et al., 2011; Talbot et al., 2012). The inability to respond to insulin and IGF leads to brain “starvation” and neuronal loss (de la Monte et al., 2009; de la Monte, 2012). Moreover, reducing the activity of the insulin/IGF signaling cascade seems to protect from AD-like neurodegeneration in nematodes, possibly by promoting more densely packed (and less toxic) amyloid fibrils (Cohen and Goedert, 2004; El-Ami et al., 2014). Thus, the link between AD and insulin/IGF exists, but it is not easy to decipher. However, some of the mechanisms involved are becoming clear. For example, the kinases that promote Tau phosphorylation, causing cell death, become increasingly activated due to insulin resistance (Schubert et al., 2003, 2004). Then, Aβ-42 and its precursor protein levels also increase in the brain as a result of insulin resistance (Messier and Teutenberg, 2005). One can state that, what could be called the “brain diabetes phenotype,” i.e., increased resistance to insulin and to IGF, can result in the appearance of classical AD molecular biomarkers. Besides these clear links between diabetes and AD-related peptides and proteins, the physiological functioning of insulin and IGF promotes neuronal growth, differentiation, and the formation of synapses, the lack of which is associated with dementia (Takeda et al., 2010; Westwood et al., 2014). Overall, insulin and IGF are required for synaptic plasticity and are necessary for the cognitive function, the mechanisms of which are only partially explained (Qiu et al., 1998; Wickelgren, 1998; Zhao and Alkon, 2001). Oxidative stress is also associated with AD and diabetes as well as advanced glycation end products (Ramasamy et al., 2011; Silveira et al., 2019).

Although studies focusing on IAPP, insulin, and IGF are stimulating and may lead to exciting developments, one must be careful to draw definitive conclusions regarding multi-factorial diseases such as AD, even if it has been analyzed through the prism of the glucose metabolism. The road to a treatment for AD is full of failed starts and drug-development pipeline failures even if one (partially) understands the mechanism involved (Berhanu et al., 2013). The fact that aging implies reductions in insulin and IAPP release (Dechenes et al., 1998) provides important clues that, in retrospect, should not have been overlooked for so long (Despa and Decarli, 2013). The most powerful process may be related to IGF-I, which has been shown to protect and rescue hippocampal neurons from Aβ-42 neurotoxicity and IAPP-induced toxicity, as a two-in-one solution. This was already reported over 20 years ago (Doré et al., 1997), but, inexplicably, it was somewhat ignored. This is no longer the case: the role of IAPP in AD is not overlooked, as IAPP is even seen as the second amyloid of AD pathology, a promising approach to understand IAPP in relation to AD (Fawver et al., 2014). A curious finding is that Aβ-42 directly activates the amylin-3 receptor subtype, which may have major implications in AD pathology (Fu et al., 2012) as well as in the “brain diabetes phenotype” that we have proposed here. Moreover, it may also explain why pramlintide, which acts on rat and human amylin receptors (Gingell et al., 2014), can be protective in AD. Interestingly, Aβ-42 expressed on human neurons can bind to amylin receptors (Jhamandas et al., 2011), thereby triggering activation of apoptotic genes, as IAPP does (Jhamandas and Mactavish, 2012). The activity of these molecules on the brain may lead to neuronal death, particularly in AD patients, thus explaining their phenotypic profiles (Kawarabayashi et al., 2001; Dubois et al., 2016; Li and Huang, 2016; Li et al., 2016a).

## Strategies for Reducing IAPP Proteotoxicity Using Natural Compounds

The links between IAPP and AD have not gone unnoticed, with some authors presenting relevant reviews on the topic and hinting at possible therapeutic strategies (Despa and Decarli, 2013; Jackson et al., 2013; Bharadwaj et al., 2017; Mietlicki-Baase, 2018). The role of IAPP is undeniably relevant in both diabetes and AD. Therefore, attempting to modulate the oligomerization process or block its cytotoxicity is an appealing venue for therapeutic strategies. Different approaches have been attempted to block protein aggregation (Figure 1C). Efforts have been made to interfere with the oligomerization process itself by (i) stabilizing the monomer, (ii) remodeling small oligomers from a fibrillogenic to non-fibrillogenic form, thereby creating “off-pathway” oligomers, and (iii) reverting fibrils to monomers or other intermediate species (Pithadia et al., 2016; Table 1). Another strategy is to revert the pathological effects of oligomers in cellular homeostasis, such as ER stress, mitochondrial damage, cell membrane permeabilization, autophagy impairment, inflammation, and β-cell death (Kiriyama and Nochi, 2018).

**Table 1:** Effect of (poly)phenols on the aggregation of human IAPP.

**Phenolic compound**	**Experimental model**	**Mechanism of action**	**References**
**Baicalein** 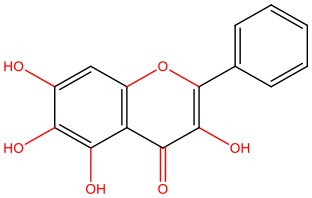	• Cell-free	• Inhibits the formation of β-sheet structures	Mirhashemi, 2012
	• Cell-free	• Inhibits IAPP amyloid formation	Velander et al., 2016
	• INS-1 rat pancreatic β-cell line exposed to hIAPP aggregates	• Neutralizes IAPP-induced cytotoxicity in a dose depend manner	
**Curcumin** 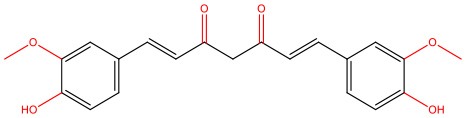	• Cell-free	• Modulates IAPP self-assembly by unfolding α-helix structures	Sparks et al., 2012
	• Cell-free	• Induces the dissociation of amyloid fibrils	Shoval et al., 2008
	• Cell-free	• Alters the morphology and conformation of IAPP aggregates	Daval et al., 2010
	• INS-1 rat pancreatic β-cell line exposed to hIAPP aggregates	• Protects cells against amyloid-induced toxicity	
**ECG** 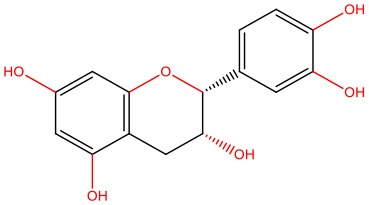	• Cell-free	• Reduces the rate constants of first nucleation step of amyloid fibril formation, inhibiting the first stages of this process	Kamihira-Ishijima et al., 2012
**EGCG** 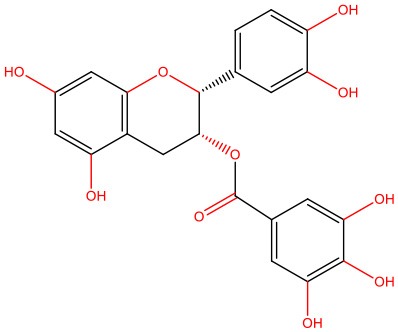	• Cell-free	• Binds to specific conformers within an ensemble of IAPP monomers, affecting the oligomerization process and fibril assembly	Young et al., 2014a
	• Cell-free	• Delays the formation of β-sheet containing IAPP aggregates • Stabilizes non-fibrillar large aggregates during fibrillogenesis	Suzuki et al., 2012
	• Cell-free	• Inhibits the formation of IAPP-NH_2_ fibrils • Promotes the generation of IAPP-NH_2_ amorphous aggregates	Xu et al., 2017
	• Cell-free	• Remodels IAPP fibrils, but does not fully resolubilize them to unstructured monomers	Cao and Raleigh, 2012
	• Cell-free	• Presents an amyloid remodeling activity that is dependent on its auto-oxidation	Palhano et al., 2013
	• Cell-free	• Destabilizes IAPP oligomers • Breaks the initial ordered pattern of two polymers, decreases their β-sheet content, and enlarges their conformational space	Wang et al., 2014
	• Cell-free	• Acts as an efficient amyloid inhibitor, especially in bulk solution • Does not disaggregate amyloid fibrils at a phospholipid interface	Engel et al., 2012
	• Cell-free	• Binds to IAPP and induces the formation of amorphous aggregates	Franko et al., 2018
	• Cell-free	• Disaggregates preformed amyloid fibrils derived from IAPP	Meng et al., 2010
	• INS-1 rat pancreatic β-cell line exposed to hIAPP aggregates	• Protect cells against IAPP-induced cytotoxicity	
	• RIPHAT transgenic mice expressing hIAPP (sub-chronic administration)	• Reduces the amount of IAPP fibrils in the pancreas but does not alter the disease clinical signs	Franko et al., 2018
**EGCG/Al(III)**	• Cell-free	• Inhibits IAPP fibrillation	Xu et al., 2016
**EGCG:Zn(II) complex**	• Cell-free	• Suppresses IAPP amyloid aggregation, both in the presence and absence of a lipid membranes • Promotes the stabilization of a helical structure of IAPP	Lee et al., 2019
	• RIN-5F rat pancreatic β-cell line exposed to hIAPP aggregates	• Suppresses the cellular toxicity mediated by IAPP	
**Ferulic acid** 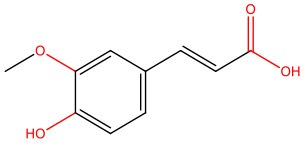	• Cell-free	• Represses IAPP amyloid formation	Mirhashemi, 2012
**Fisetin** 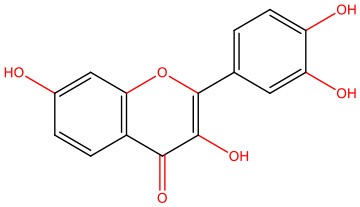	• Cell-free	• Inhibits the formation of β-sheet structures	Aarabi and Mirhashemi, 2017
**Genistein** 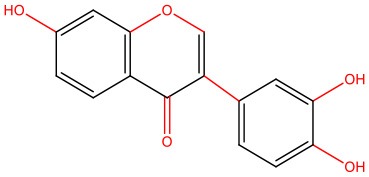	• Cell-free	• Prevents the conformational transition of IAPP monomers to β-sheet structures • Decreases amyloid fibrillization • Interferes with self-aggregation of IAPP oligomers	Ren et al., 2018
	• RIN-5F rat pancreatic β-cell line exposed to hIAPP aggregates	• Reduces IAPP cytotoxicity • Increases cell viability, decreases cell apoptosis, and reduces cell membrane leakage	
**Morin** 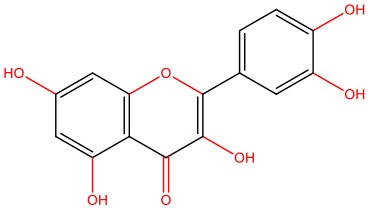	• Cell-free	• Inhibits the generation of IAPP aggregates • Promotes the disaggregation of preformed fibrils • Inhibits insulin aggregation and prevents conformational changes	Noor et al., 2012
	• Cell-free	• Changes the morphology, solvent accessible surface area, and the secondary structure of IAPP pentamer	Wang et al., 2015b
**Myricetin** 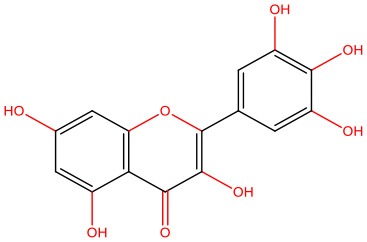	• Cell-free	• Inhibits IAPP fibrillogenesis	Zelus et al., 2012
	• PC12 rat adrenal gland cell line exposed to hIAPP aggregates	• Reduces IAPP-induced cytotoxicity	
**O4, orcein-related small molecule** 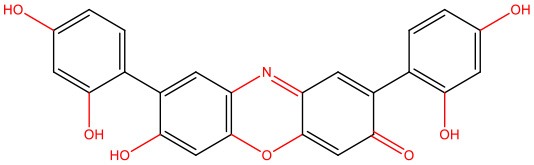	• Cell-like system (using artificial crowding agents Ficoll 70 and sucrose)	• Generates globular, amorphous off-pathway assemblies, inhibiting the polymerization of mature IAPP fibrils	Gao et al., 2015
**Oleuropein aglycone** 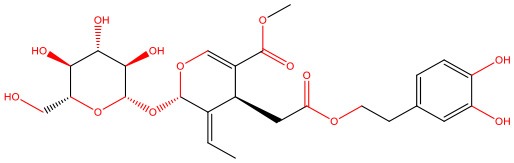	• Cell-free	• Favors the generation of off-pathway IAPP species	Rigacci et al., 2010
	• RIN-5F rat pancreatic β-cell line exposed to hIAPP aggregates	• Reduces IAPP cytotoxicity	
	• INS-1 rat pancreatic β-cell line exposed to hIAPP aggregates	• Promotes glucose-stimulated insulin secretion • Stimulates the ERK/MAPK signaling pathway • Inhibits the cytotoxicity mediated by IAPP amyloids	Wu et al., 2017
**PGG** 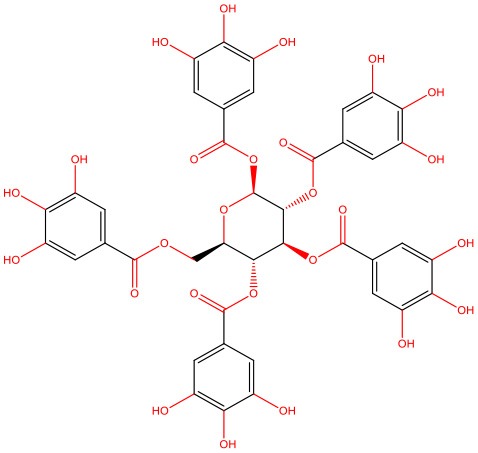	• Cell-free	• Inhibits IAPP aggregation and amyloid-based fiber formation	Bruno et al., 2013
	• PC12 rat adrenal gland cell line exposed to hIAPP aggregates	• Prevents the toxicity of IAPP oligomers	
**Quercetin** 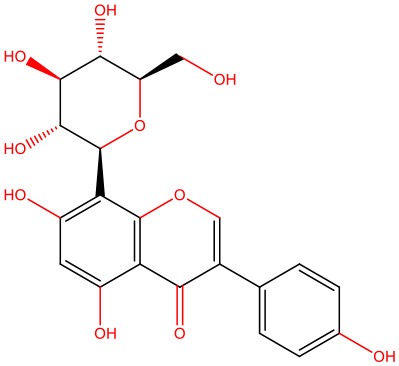	• RIN-5F rat pancreatic β-cell line exposed to hIAPP aggregates	• Modulates the aggregation propensity of IAPP • Protects cells from IAPP cytotoxicity • Reduces oxidative damage	López et al., 2016
**Resveratrol** 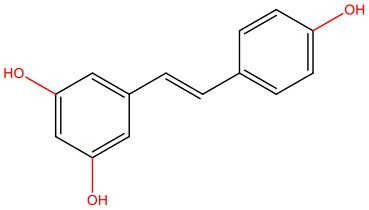	• Cell-free	• Stabilizes IAPP off-pathway oligomers	Nedumpully-Govindan et al., 2016
	• Cell-free	• Inhibits the stacking of IAPP oligomers, avoiding its aggregation and accumulation	Jiang et al., 2011
	• Cell-free	• Promotes conformational changes of hIAPP1 pentamer (alters secondary structures, order degree, and morphology)	Wang et al., 2015a
	• Cell-free	• Inhibits IAPP aggregation in the presence of aggregation-fostering negatively charged lipid interfaces	Evers et al., 2009
	• POPG model membrane	• Promotes the generation of secondary structures (sheets and helices) • Perturbs the interaction between IAPP and negative charged membranes	Lolicato et al., 2015
	• INS-1 rat pancreatic β-cell line exposed to hIAPP aggregates	• Arrests IAPP fibril generation and associated cytotoxic effects at an early stage	Radovan et al., 2009
	• INS-1 rat pancreatic β-cell line exposed to hIAPP aggregates	• Generates off-pathway non-toxic IAPP conformations • Enhances cell survival	Mishra et al., 2009
	• INS-1 rat pancreatic β-cell line expressing hIAPP	• Decreases amyloid deposition and restores insulin secretion, though only when autophagy is not blocked	Lv et al., 2019
**Resveratrol derivate**	• POPC/POPS model membrane	• Eliminates amyloid growth and associated-membrane damage	Sciacca et al., 2018
**Rosmarinic acid** 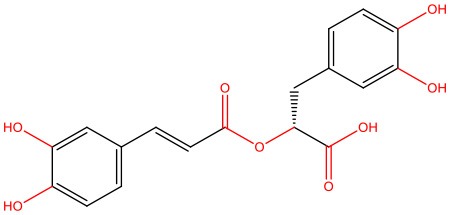	• Cell-free	• Represses IAPP amyloidogenic aggregates by opening the β-sheet conformation of these structures • Reduces IAPP-mediated toxicity	Zheng and Lazo, 2018
**Rutin** 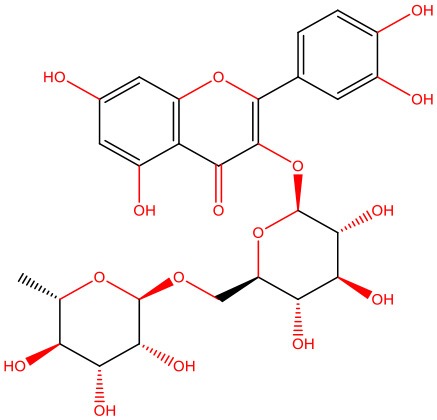	• Cell-free	• Inhibits IAPP misfolding, disaggregates IAPP oligomers and reverts IAPP conformation toward the physiological state	Aitken et al., 2017
	• FVB/NJ transgenic mice expressing hIAPP	• Slows diabetes progression	
	• SH-SY5Y human neuroblastoma cell line exposed to hIAPP aggregates	• Inhibits IAPP aggregation and reduces IAPP-induced neurotoxicity and oxidative stress • Reduces the production of ROS and NO • Attenuates mitochondrial damage	Yu et al., 2015
	• BV-2 mouse microglial cell line exposed to hIAPP aggregates	• Inhibits IAPP aggregation and reduces IAPP-induced neurotoxicity • Increases GSH/GSSG ratio • Reduces the production of MDA, GSSG and pro-inflammatory cytokines (TNF-α and IL-1β)	
**Salvianolic acid B** 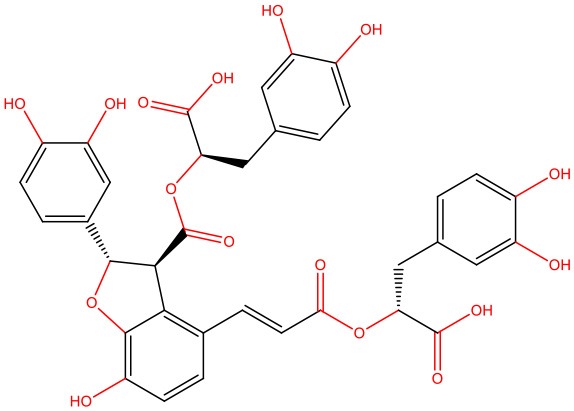	• INS-1 rat pancreatic β-cell line exposed to hIAPP aggregates	• Suppresses membrane permeabilization, mitochondrial impairment, and cytotoxicity induced by IAPP • Inhibits the formation of lower order oligomers and fibrils	Cheng et al., 2013
**Silibinin** 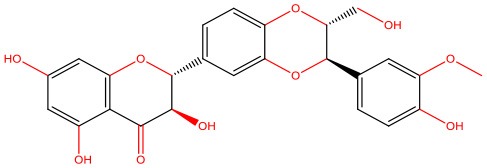	• Cell-free	• Binds to specific conformers within an ensemble of IAPP monomers, affecting the oligomerization process and fibril assembly	Young et al., 2014a
	• Cell-free	• Favors the 3+ IAPP monomer preventing oligomerization • Disaggregates preformed fibrils into small off-pathway oligomers	Young et al., 2014b
	• Cell-free	• Inhibits IAPP fibrillization through the suppression of toxic IAPP oligomerization	Cheng et al., 2012
	• INS-1 rat pancreatic β-cell line exposed to hIAPP aggregates	• Reduces IAPP cytotoxicity in a dose-dependent manner	
	• INS-1 rat pancreatic β-cell line exposed to hIAPP aggregates	• Enhances estrogen receptors phosphorylation, leading to downregulation of ROS/RNS production induced by IAPP/Aβ-42	Yang et al., 2019a
	• INS-1 rat pancreatic β-cell line exposed to hIAPP aggregates	• Protects cells from IAPP-induced apoptosis through activation of GLP-1R/PKA signaling	Yang et al., 2019b
**8-β-d-Glucopyranosylgenistein** 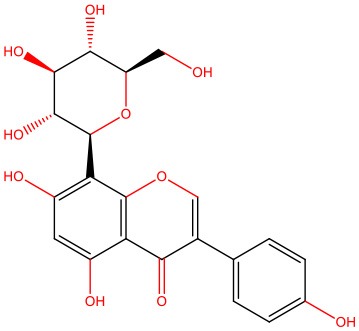	• Cell-free	• Interacts with IAPP oligomers, preventing amyloid fibrillization	Jesus et al., 2014
	• STZ-induced diabetic rats	• Normalizes fasting hyperglycemia • Ameliorates excessive post-prandial glucose excursions • Increases β-cell sensitivity and insulin secretion	

*Al(III), Aluminum III; Aβ, Amyloid beta; ECG, Epicatechin-3-Gallate; EGCG, Epigallocatechin-3-Gallate; ERK, Extracellular-Signal-Regulated Kinase; FVB/NJ, Friend Virus B NIH Jackson; GLP-1R, Glucagon-like Peptide-1 Receptor; GSH, Glutathione; GSSG, Glutathione disulfide; hIAPP, Human Islet Amyloid Polypeptide; hIAPP-NH_2_, Amidated Human Islet Amyloid Poplypeptide; IL-1β, Interleukin-1beta; MAPK, Mitogen Activated Protein Kinase; MDA, Malondialdehyde; NO, Nitric Oxide; PGG, Pentagalloyl Glucose; PKA, Protein Kinase A; POPC, 2-oleoyl-1-pamlitoyl-sn-glyecro-3-phosphocholine; POPG, 2-oleoyl-1-pamlitoyl-sn-glyecro-3-glycerol; POPS, 1-palmitoyl-2-oleoyl-sn-glycero-3-phospho-l-serine; RNS, Reactive Nitrogen Species; ROS, Reactive Oxygen Species; STZ-induced diabetic rat, Streptozotocin-induced diabetic rat; TNF-α, Tumor Necrosis Factor-alpha; Zn(II), Zinc II*.

The pleiotropic action of (poly)phenols toward chronic diseases, particularly diabetes, is well-documented (Bahadoran et al., 2013; Panickar, 2013; Jasmin and Jaitak, 2019; Silveira et al., 2019). Most importantly, (poly)phenols have been linked to the inhibition of aggregation of proteins such as IAPP and Aβ-42 (Pithadia et al., 2016; Sequeira and Poppitt, 2017; Dhouafli et al., 2018). It has been shown that different classes of (poly)phenols may interfere with different steps of the oligomerization process (Ladiwala et al., 2011). The lower toxicity of these compounds compared to synthetic molecules gives them an advantage as future therapeutics. However, there is an urgent need for the validation of their therapeutic potential in pre-clinical studies, as most of the evidences derives from cell-free and *in vitro* assays (Table 1).

Epigallocatechin gallate (EGCG) and resveratrol are the most-studied compounds. EGCG has been proved to remodel IAPP oligomers, create “off-pathway” intermediates, and prevent monomers from shifting into β-sheet structures, a critical step in early-stage aggregation processes (Bieschke et al., 2010; Young et al., 2014a; Nedumpully-Govindan et al., 2016). Resveratrol has also been suggested as an inhibitor of both IAPP and Aβ-42 pathological effects. It was reported to lower intracellular and secreted levels of Aβ-42 and also to stimulate intracellular degradation (Marambaud et al., 2005). However, resveratrol seems to be less effective than EGCG and inefficient in preventing amyloid formation (Tu et al., 2015). In addition, (poly)phenols have an important role in reducing oligomer-induced cytotoxicity by modulating oxidative stress (Chakrabarti et al., 2013), inflammation (Apetz et al., 2014), and autophagy (Rigacci et al., 2015). A compilation of (poly)phenols as bioactive components modulating IAPP toxicity is given in Table 1.

## Concluding Remarks

This study shows how an “old story” can originate ground-breaking knowledge and create new venues for a therapeutic approach. The first high-impact paper describing IAPP as a relevant factor for T2DM was published in 1994 (Lorenzo et al., 1994). Since then, even though it took a long time for this field to be pursued, knowledge has come a long way. It is now clear that direct brain microvascular injury, leading to white matter disease, is unequivocally originated by elevated IAPP levels in diabetes (Ly et al., 2017), further supporting the “diabetes brain phenotype” hypothesis that we have proposed here.

This change of approach is as cutting-edge as the finding that amyloid fibrils precursors, but not the amyloid fibrils themselves, are the cause of toxicity (Martins et al., 2008). We believe that this study, and others that reflect on the role of IAPP in AD in an unbiased manner (Mietlicki-Baase, 2018) complemented by further experiments, will certainly pave the road to future IAPP-centered drug development strategies against AD, as we considering it as the result of a “diabetes brain phenotype.” Such a view will certainly yield major therapeutic advances.

## Author Contributions

AR and SF wrote the manuscript. IM wrote and revised the manuscript. RM designed the layout and wrote and revised the manuscript.

### Conflict of Interest

The authors declare that the research was conducted in the absence of any commercial or financial relationships that could be construed as a potential conflict of interest.
